# Reading program research proposal

**DOI:** 10.1192/j.eurpsy.2021.598

**Published:** 2021-08-13

**Authors:** D.M.S. Abbasy, D.R. Cruz, A. Fitzgerald

**Affiliations:** Child And Adolescent Psychiatry, RUSH University Medical Center, Chicago, United States of America

**Keywords:** Specific Learning Disorder, Reading Program, child and adolescent psychiatry, Reading

## Abstract

**Introduction:**

There is a significant problem of unidentified and unaddressed reading disabilities leading to psychiatric problems in children and adolescents because of not having proper tools of assessments in schools. This research proposal can be a revolutionary paradigm in identifying, classifying, modeling, and benefitting children and adolescents with a specific learning disorder (SLD).

**Objectives:**

The objective of the current research proposal is to provide a framework of our reading program and collect data over time as cohorts to reflect the positive outcomes of the reading program.

**Table:** 

Sub-Objectives
1. To provide an intervention that is accessible and feasible for children and their parents that will improve their academic and socio-emotional aspects.
2. Educate parents regarding SLD.
3. To provide reading training to address SLD in reading and improve reading.
4. To provide Cognitive Behavioral Therapy (CBT) to target the anxiety and depression that results because of having a SLDin reading.

**Methods:**

After a reading assessment, students with specific reading disabilities will be registered in the program for 10 weeks. Every student will have reading training and CBT on different days of the week via video conference. Data will be collected retrospectively from the initial cohort and subsequent cohorts will be added to the data collection process for a final analysis when 60 students have completed the program.

**Results:**

Initial two weeks of reading training and CBT shows positive and promising results so far.

**Conclusions:**

Children need to be screened at a young age for a reading disability before they struggle academically, and develop psychiatric issues later in life.

**Conflict of interest:**

The aim of this research proposal is to help us understand, evaluate and benefit children with Specific Learning Disorder (SLD) with our newly setup reading program at RUSH University Medical Center, Department of Child and Adolescent Psychiatry.

